# Purification of chicken carbonic anhydrase isozyme-III (CA-III) and its measurement in White Leghorn chickens

**DOI:** 10.1186/1751-0147-53-63

**Published:** 2011-11-26

**Authors:** Toshiho Nishita, Yuichiro Tomita, Daisuke Yorifuji, Kensuke Orito, Hideharu Ochiai, Kazuyosi Arishima

**Affiliations:** 1 Laboratories of Veterinary Physiology 1, School of Veterinary Medicine, Azabu, University,1-17-71 Fuchinobe, Sagamihara, Kanagawa Japan 252-5201; 2 Isogaya Yokeien, Kamiishigami, Otawara-shi, Tochigi-ken, Japan 324-0037; 3 Laboratories of Veterinary Physiology 2, School of Veterinary Medicine, Azabu, University,1-17-71 Fuchinobe, Sagamihara, Kanagawa, Japan 252-5201; 4 Research Institute of Biosciences, Azabu University, 1-17-71, Fuchinobe, Sagamihara, Kanagawa, Japan 252-5201; 5 Veterinary Anatomy 2, School of Veterinary Medicine, Azabu University, 1-17-71, Fuchinobe, Sagamihara, Kanagawa, Japan 252-5201

## Abstract

**Background:**

The developmental profile of chicken carbonic anhydrase-III (CA-III) blood levels has not been previously determined or reported. We isolated CA-III from chicken muscle and investigated age-related changes in the levels of CA-III in blood.

**Methods:**

CA-III was purified from chicken muscle. The levels of CA-III in plasma and erythrocytes from 278 female chickens (aged 1-93 weeks) and 68 male chickens (aged 3-59 weeks) were determined by ELISA.

**Results:**

The mean level of CA-III in female chicken erythrocytes (1 week old) was 4.6 μg/g of Hb, and the CA-III level did not change until 16 weeks of age. The level then increased until 63 weeks of age (11.8 μg/g of Hb), decreased to 4.7 μg/g of Hb at 73 weeks of age, and increased again until 93 weeks of age (8.6 μg/g of Hb). The mean level of CA-III in erythrocytes from male chickens (3 weeks old) was 2.4 μg/g of Hb, and this level remained steady until 59 weeks of age. The mean plasma level of CA-III in 1-week-old female chickens was 60 ng/mL, and this level was increased at 3 weeks of age (141 ng/mL) and then remained steady until 80 weeks of age (122 ng/mL). The mean plasma level of CA-III in 3-week-old male chickens was 58 ng/mL, and this level remained steady until 59 weeks of age.

**Conclusion:**

We observed both developmental changes and sex differences in CA-III concentrations in White Leghorn (WL) chicken erythrocytes and plasma. Simple linear regression analysis showed a significant association between the erythrocyte CA-III level and egg-laying rate in WL-chickens 16-63 weeks of age (p < 0.01).

## Background

Carbonic anhydrase (CA; EC 4.2.1.1), a well-characterized enzyme, catalyzes the reversible hydration of CO_2 _to form HCO_3_^- ^and protons according to the following reaction: CO_2 _+ H_2_O ↔ HCO_3_^- ^+ H^+^. The enzyme can (reversibly) convert carbon dioxide into bicarbonate and hydrogen ions without the formation of carbonic acid. The first reaction is catalyzed by carbonic anhydrase and the second occurs instantaneously. CA plays important roles in gas transport, acid/base regulation, bone resorption and calcification, ion transport, and various secretory functions in several tissues [1]. There are at least 11 active CA isozymes (cytosolic CA-I, CA-II, CA-III, and CA-VII, mitochondrial CA-V_A _and V_B_, secretory CA-VI, and membrane-associated CA-IV, IX, XII, and XIV), as well as 3 inactive CA-related proteins (CA-VIII, X, and XI) [2,3]. Each isozyme has a unique molecular structure. CA-III has been demonstrated in human [4], horse [5], cow [6], sheep [7], rabbit [8], dog [9], pig [10], and chicken [7] skeletal muscle. Among the isozymes, CA-III has the lowest turnover rate and greatest resistance to inhibition by sulphonamides. CA-III may facilitate the transport of CO_2 _in skeletal muscle by catalyzing the reversible hydration of CO_2 _to the more soluble HCO_3_^-^. Raisanen et al. [11] suggested that CA-III is an oxyradical scavenger and thus protects cells from oxidative damage. CA-III is relatively specific to skeletal muscle [4,12] and could thus be useful as a diagnostic marker for muscle disease [13,14].

We found no previous reports on CA-III blood concentrations in healthy chickens among the studies on chicken CA-III published thus far. The present study aimed to establish simple methods for the purification of chicken CA-III and develop an enzyme-linked immunoassay (ELISA) system to measure CA-III levels in erythrocytes and plasma from normal WL chickens.

## Methods

### Purification of chicken CA-III

The leg muscles were excised from several female white Leghorn (WL) chickens killed by an overdose of Nembutal. WL-chickens. Sample of muscle, weighing 200 g, was homogenized at 4°C in a blender containing 400 mL of 0.01 *M *Tris-HCl buffer (pH 8.0). All experiments were performed according to the guidelines of The Laboratory Animal Care Committee of Azabu University and in compliance with The Japanese Animal Welfare Guide. The homogenate was centrifuged at 27,000 × g for 30 min at 4°C. Iodoacetamide was then added to a final concentration of 0.01 *M*, the pH of the solution was adjusted to 8.0, and the mixture was incubated for 30 min under non-denaturing conditions. The sample was dialyzed against 0.01 *M *2-(*N*-morpholino) ethanesulfonic acid (MES) buffer (pH 6.0) (Dojindo Laboratories, Kumamoto, Japan) at 4°C. The dialyzed sample was centrifuged at 27,000 × g for 30 min at 4°C, and the supernatant was applied to a carboxymethyl (CM)-cellulose column (CM 52, Whatman International Ltd., Maidstone, UK) (3.4 × 30 cm) equilibrated with 0.01 *M *MES buffer (pH 6.0). After extensive washing, adsorbed proteins were eluted with a linear gradient of 0-0.15 *M *NaCl in 0.01 *M *MES buffer (pH 6.0) at a flow rate of 20 mL/h using a peristaltic pump. The eluate was collected in 6 mL fractions. The optical densities at 280 nm of all fractions were recorded. Fractions with enzymatic activity were collected and pooled. Pooled samples were precipitated with saturated ammonium sulfate, and the precipitate was collected by centrifugation. Precipitates were then dissolved in a small amount of distilled water. Samples were next passed at a flow rate of 15 mL/h over a Sephacryl S-200 HR (Sephacryl HR, Pharmacia Biotech, Uppsala, Sweden) column equilibrated with 0.05 *M *Tris-HCl (pH 8.0) containing 0.5 *M *NaCl. The major peak had CA activity and was collected and dialyzed against water. Samples were further purified by the column electrofocusing method of Svenson [15] using an electrofocusing column and Ampholine (pH 8.0-10.5, Pharmacia Biotech, Uppsala, Sweden). The fraction containing CA activity was dialyzed against 0.01 *M *Tris-HCl buffer (pH 7.5). Purified chicken CA-III was stored at -80°C until use.

### Characterization of purified chicken CA-III

#### Partial amino acid sequencing of chicken CA-III

Purified chicken CA-III was cleaved with endoproteinase Glu-C (Sigma-Aldrich Corp., St. Louis, MO, USA), in the presence of sodium dodecyl sulfate (SDS), according to the Cleveland method [16]. Digested chicken CA-III was separated by sodium dodecyl sulfate polyacrylamide gel electrophoresis (SDS-PAGE). One of the main peptide bands was sequenced using a Procise 491 protein sequencer (Applied Biosystems, Foster City, CA, USA) to obtain a partial amino acid sequence.

### Electrophoretic procedures and western blotting

SDS-PAGE was performed using 12.5% PhastGel Homogeneous gels and the PhastSystem (Pharmacia Biotech, Uppsala, Sweden). Thin-layer isoelectric focusing (IEF) was performed with PhastGel IEF media and the PhastSystem. A low molecular weight calibration kit and isoelectric point calibration kit (Pharmacia Biotech) were used to determine the molecular weight and isoelectric point of the protein. After electrophoresis, the gel was stained with Coomassie Brilliant Blue.

Western blotting was performed as previously described [17]. Briefly, adequate volumes of hemolysate and purified chicken CA-III were separated on 12.5% PhastGel Homogeneous gels and transferred to Immobilon PVDF transfer membrane (Millipore Corp, Bedford, Mass, USA) using a commercially available transfer system. The buffer for electrophoretic transfer contained 25 m*M *Tris (pH 8.3), 192 m*M *glycine, 0.1% SDS, and 15% methanol. CA-III was detected using rabbit anti-chicken CA-III antiserum produced in the present study. This antiserum was diluted 1:2000 in 5 mL of 50 m*M *Tris-HCl (pH 7.5) containing 0.3% BSA, 0.9% NaCl, 0.01% thimerosal, and 10 m*M *EDTA (buffer A). After incubation with the primary antibody, membranes were washed in 0.15 *M *phosphate-buffered saline solution (PBS) containing 0.05% Tween (PBS-Tween) and incubated with peroxidase-conjugated goat anti-rabbit immunoglobulin G (IgG) antiserum (Kirkegaard & Perry Laboratories Inc., Gaithersburg, CA, USA) diluted 1:4,000 in 5 mL of buffer A. Membranes were washed again with PBS-Tween and developed by incubation with 0.02% hydrogen peroxide and 0.2 m*M *diaminobentidine-tetrahydrochloride in 0.05 *M *Tris-HCl (pH 7.6) for approximately 5 min.

### Enzyme analysis

The enzymatic activity of CA was measured using the method of Wilbur and Anderson [18]. Assays were performed at 4°C, and specific activity (U) was determined according to the formula:

U=10×(Tb∕Te-1)∕mgofprotein

Where Tb is the time required for the reaction (i.e., pH change from 8.5 to 6.5) in the absence of enzyme and Te is the time required for the same reaction in the presence of enzyme. The reaction time was measured with a stopwatch.

### Immunochemistry

Antibodies against purified chicken CA-III were produced in rabbits. Each rabbit was immunized initially with 1 mg of purified CA-III emulsified with an equal volume of Freund's complete adjuvant. This was followed by a booster injection of an equivalent amount of enzyme once a week for 5 successive weeks. Ten days after the final injection, blood was obtained from the auricular vein. The specificity of the antiserum was examined by a double-immunodiffusion method and ELISA. The cross-reactivity of anti-chicken CA-III for chicken CA-II, canine CA-I, and canine CA-III was examined by ELISA. Chicken CA-II [19], canine CA-I [20], and canine CA-III [9] had been previously purified. Immunoglobulin G (IgG) was purified using Protein A.

### Biotinylation of chicken CA-III

A 2-mL volume of a solution containing 5 mg/mL purified chicken CA-III was incubated for 4 h at 25°C with 4.54 mg of biotin (sulfosuccinimidyl N-[N-(D-biotinyl)-6-aminohexanoyl]-6-aminohexanoate; Dojindo Laboratories, Kumamoto, Japan) in 0.08 mL of 10 m*M *HEPES buffer (pH 8.5) (Dojindo Laboratories). Conjugates were then were dialyzed extensively against PBS (pH 7.5).

### Blood samples

Blood samples of clinically normal male WL-chickens (SPF, Line M) at 3 (n = 9), 10 (n = 9), 12 (n = 10), 17 (n = 10), 20 (n = 10), 27 (n = 5), 30 (n = 5), 52 (n = 5), and 59 (n = 5) weeks of age were purchased from Nisseiken Co., Ltd. (Tokyo, Japan). Blood samples obtained from clinically normal female WL-chickens (LOHMAN LSL-LITE) at 1 (n = 20), 3 (n = 20), 9 (n = 19), 12 (n = 20), 16 (n = 20), 21 (n = 20), 25 (n = 20), 31 (n = 20), 49 (n = 19), 63 (n = 20), 69 (n = 20), 73 (n = 20), 80 (n = 20), and 93 (n = 20) weeks of age were provided by egg farm (Isogaya Yokeien, Tochigi, Japan). WL-chickens were housed in a conventional curtain side wall cage house with four hens per cage. The forced molt was induced at 64 and finished at 67 weeks of age of all female WL-chickens by the method to reduce quantity and a calorie of the diet. The lighting hours for the force molted group was reduced to 8 hours.

Blood samples were take from individuals of different ages at the same occasion. Blood samples were mixed with lithium heparin and centrifuged at 4,500 × g for 15 min at 4°C to separate plasma from erythrocytes. Erythrocytes were lysed with an equal volume of distilled water and centrifuged at 27,000 × g for 30 min at 4°C. Hemolysates and plasma were stored at -20°C until analyzed. Plasma with evidence of hemolysis was not used for CA-III measurements. Samples (0.1 mL volume) of hemolysate at dilutions from 1:4,000 to 1:16,000 and plasma at dilutions from 1:5 to 1:10 in buffer A were subjected to ELISA in duplicate.

The egg-laying rates of WL-chicken at the ages of 21, 25, 31, 49, 63, 69, 73, 80, and 93 weeks were approximately 20, 94, 94, 95, 91, 2, 63, 91, and 82%, respectively. The egg-laying rate was 0% at the ages of 1, 3, 9, 12, and 16 weeks.

### Measurement of CA-III levels

The concentrations of CA-III in chicken hemolysates and plasma were ascertained by the competitive ELISA method. A flat-bottom micro-ELISA plate (Nunc Immuno-plate, Maxisorp, Roskilde, Denmark) was coated for 16 h at 4°C with 0.1 mL/well of anti-chicken CA-III IgG dissolved in 0.1*M *NaHCO_3 _(pH 9.6). Plates were then washed 3 times with 0.3 mL/well PBS and incubated at 23°C for 30 min with 0.2 mL/well 0.5% BSA in 0.05 *M *Tris-HCl (pH 8.0) for blocking. Each well was then washed 3 times with 0.3 mL/well PBS-Tween. The CA-III standards (6-400 ng/mL), biotinylated chicken CA-III, chicken plasma and hemolysates samples were diluted with buffer A, and ELISAs were performed in duplicate. For competition ELISAs, the biotinylated CA-III was mixed with the CA-III standards or the hemolysate and incubated for 16 h at 4°C. Each well was then washed 3 times with PBS-Tween and incubated with 0.1 mL/well of avidin and biotinylated horseradish peroxidase complex (ABC reagent, Wako Pure Chemical Industries Ltd., Tokyo, Japan) diluted 1:100 with PBS-Tween. After 30 min, each well was washed 3 times with PBS-Tween. Peroxidase activity was measured after addition of 0.1 mL/well ABTS microwell peroxidase substrate system (Kirkegaard & Perry Laboratories Inc,). The ABTS substrate system contains 2,2'-azino-di-(3-ethylbenzthiazoline-6-sulfonate) and H_2_O_2 _at a concentration of 0.3 g/L and 0.01% in a glycine/citric acid buffer, respectively. After 10 min, 0.1 mL/well of 1% SDS was added to terminate the enzyme reaction and the absorbance at 405 nm was read on an automatic ELISA reader (SH-1000, Corona Electric Co., Ltd., Ibaraki, Japan).

Several experiments were performed to optimize the assay conditions. First, the microplates were coated with several different concentrations of antibody to generate calibration curves. A concentration of 10 μg/mL coating antibody was chosen for the standard assay. The assay precision was evaluated using standard samples of 400 ng/mL, 200 ng/mL, 100 ng/mL, 50 ng/mL, 25 ng/mL, 12 ng/mL and 6 ng/mL, each assayed 5 times in 1 assay run.

### Protein assay

The concentrations of aliquots of purified chicken CA-III were determined using the Bio-Rad DC protein assay kit (Bio-Rad Laboratories, Hercules, CA, USA). Bovine serum albumin (kit catalog number 500-0111, Bio-Rad Laboratories) was used as a standard.

### Hemoglobin assay

The hemoglobin concentrations in the hemolysates were measured by the sodium lauryl sulfate-hemoglobin method using a hemoglobin B test (Wako Pure Chemical Industries Ltd., Tokyo, Japan).

### Statistical analysis

Values are expressed as means ± SD. Statistical differences in the CA-III levels of male and female chicken erythrocytes and plasma were analyzed using Student's t-test. Statistical differences in the CA-III levels of each age group were evaluated using a one-way analysis of variance (ANOVA) followed by the Bonferroni post-hoc test. Simple linear regression analysis was used to estimate the relationship between the level of CA-III and the egg-laying percentage. A significance level of p < 0.05 was used.

## Results

### Purification and characterization of chicken CA-III

**Figure 1:**
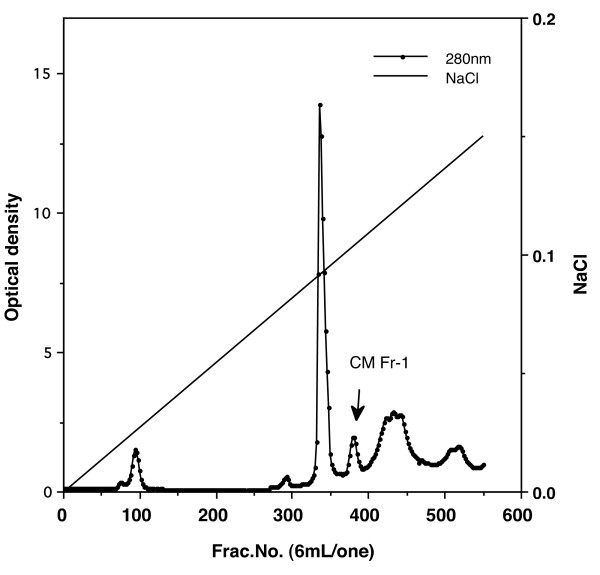
**Chromatogram illustrating elution of proteins in muscle tissue extract from a healthy chicken from a carboxymethyl cellulose column (3.4 × 30 cm) with a 0 *M *to 0.05 *M *NaCl gradient**. The total volume of the gradient was 2,500 mL. Optical density of each fraction was determined at 280 nm. The fourth peak (CM-Fr 1) of the CM-cellulose chromatography column contained CA activity.

**Figure 2:**
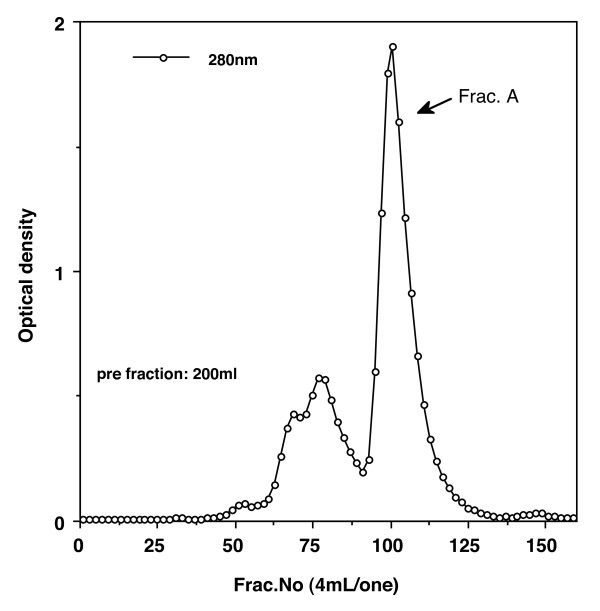
**Results of Sephacryl S-200 HR column (5.0 × 100 cm) gel filtration of the CM Fr-1 peak in Figure 1**. The main peak (Fr. A) proved to contain chicken CA-III. Starting buffer: 0.05 *M *Tris-HCl (pH 8.0) containing 0.5 *M *NaCl. Absorbance values were read at 280 nm. Flow rate: 20 mL/h.

**Figure 3:**
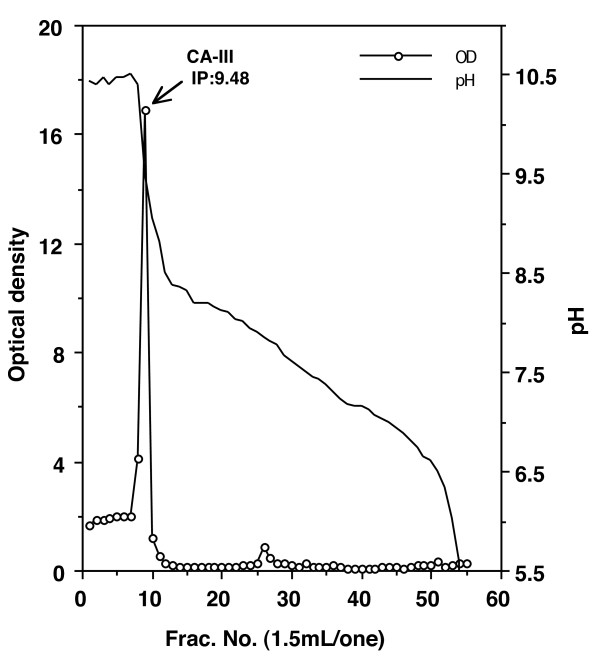
**Results of column electrofocusing of the fractions that constituted the Fr-A peak in Figure 2**. The main peak (IP: 9.48) contain chicken CA-III. Values were obtained by using a spectroscope at 280 nm. OD = optical density. Ampholine: pH 8.0-10.5.

**Figure 4:**
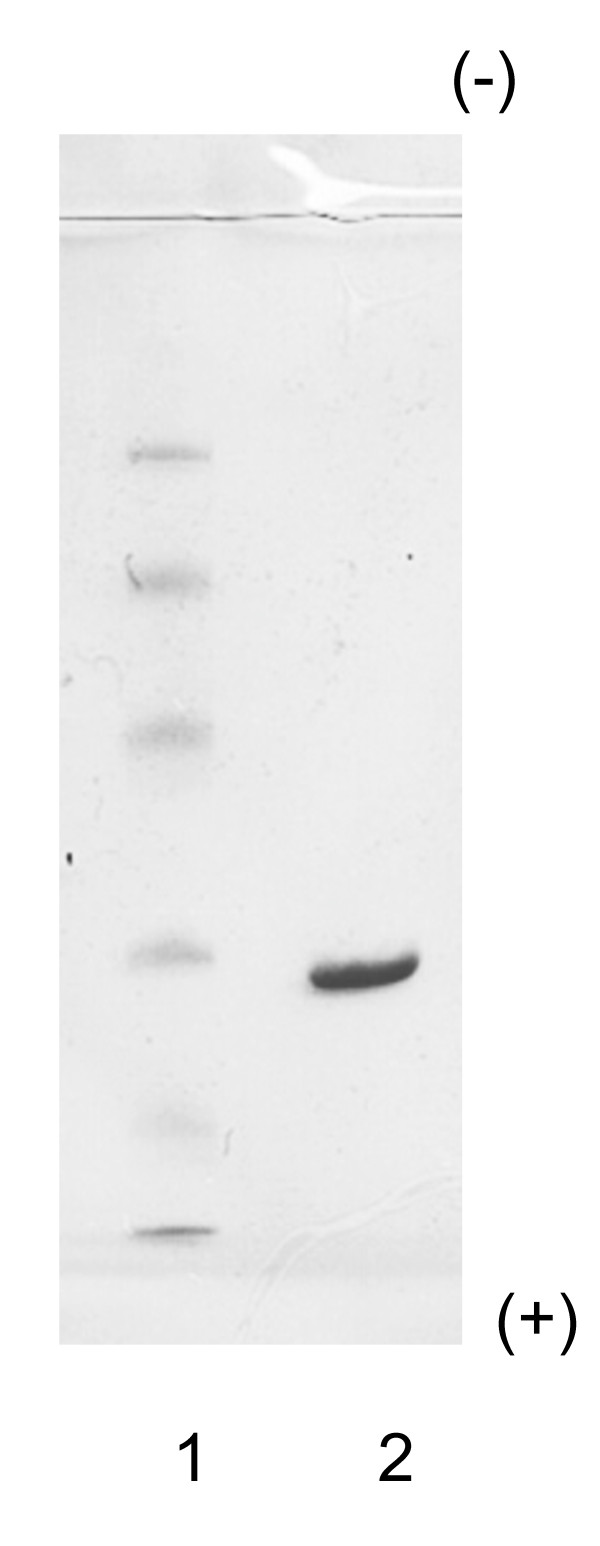
**Results of SDS-PAGE of purified chicken CA-III**. The 28 kD of purified chicken CA-III was observed. Lane 1, molecular weight marker; lane 2, purified Chicken CA-III. The low molecular weight kit contains phosphorylase (94 kD), albumin (67 kD), ovalbumin (43 kD), CA (30 kD) and trypsin inhibitor (20.1 kD).

**Figure 5:**
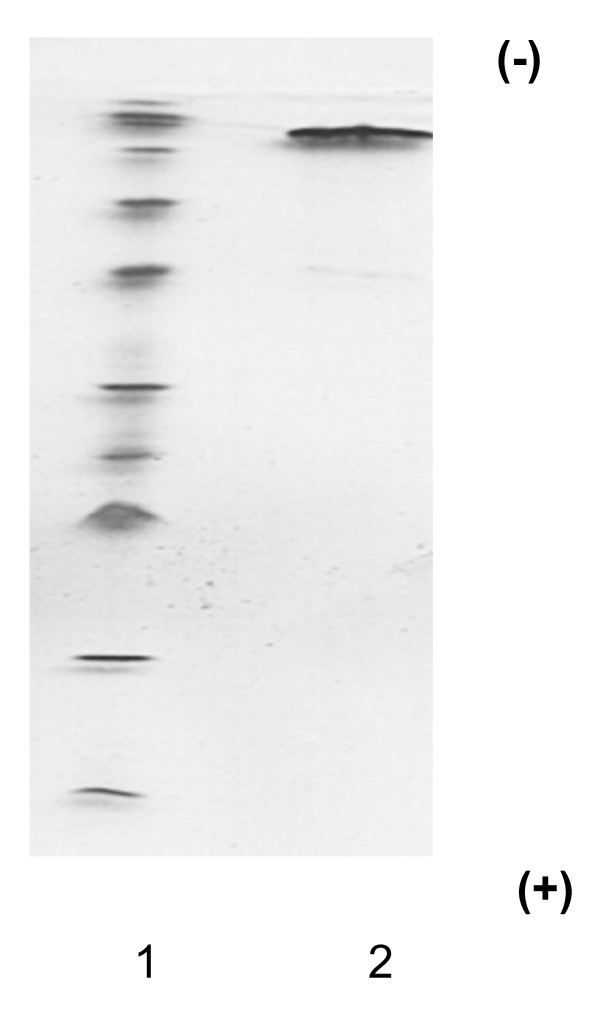
**IEF-PAGE of purified chicken CA-III**. The single bond of purified chicken CA-III was observed. Lane 1, high pI calibration markers. The high isoelectric point kit contains β-lactoglobulin A (5.20), bovine CA-B (5.85), human CA-B (6.55), horse myoglobin-acidic band (6.85), horse myoglobin-basic band (7.35), lentil lectin-acidic band (8.15), lentil lectin-middle band (8.45), lentil lectin-basic band (8.65), and trypsinogen (9.30); lane 2, chicken CA-III.

Elution profiles were plotted for each step of the purification process. The fourth peak (CM-Fr 1) of the CM-cellulose chromatography column contained CA activity (Figure 1). When fractions of CM-Fr 1 were further purified on a Sephacryl S-200 HR column, the main peak (Fr. A) proved to contain chicken CA-III (Figure 2). Figure 3 shows the column electrofocusing elution patterns of chicken CA-III. Purified chicken CA-III produced a 28 kD band by SDS-PAGE analysis (Figure 4). Purified chicken CA-III was analyzed by thin-layer isoelectric focusing (Figure 5) and produced a single band with an isoelectric point of 9.3.

A clear partial amino acid sequence (NYPMA) was obtained from digested chicken CA-III. A FASTA search of the SWISS-PROT databank revealed that this sequence was 100% identical to the carbonic anhydrase of cyanobacteria; it also showed strong similarity to the N-terminal carbonic anhydrase III of red jungle fowl (accession No. XM_418320; amino acids 23-27: NYPTA). The substitution of methionine (M) for threonine (T) may be attributable to a single nucleotide difference (ATG vs. ACG) between leghorn chickens and red jungle fowl.

The specific activity of purified chicken CA-III was 410 units/mg of protein as measured by the method of Wilbur and Anderson [18]. Therefore, we concluded that this isolated protein was carbonic anhydrase III of the White Leghorn chicken.

### Specificity of anti-chicken CA-III serum

**Figure 6:**
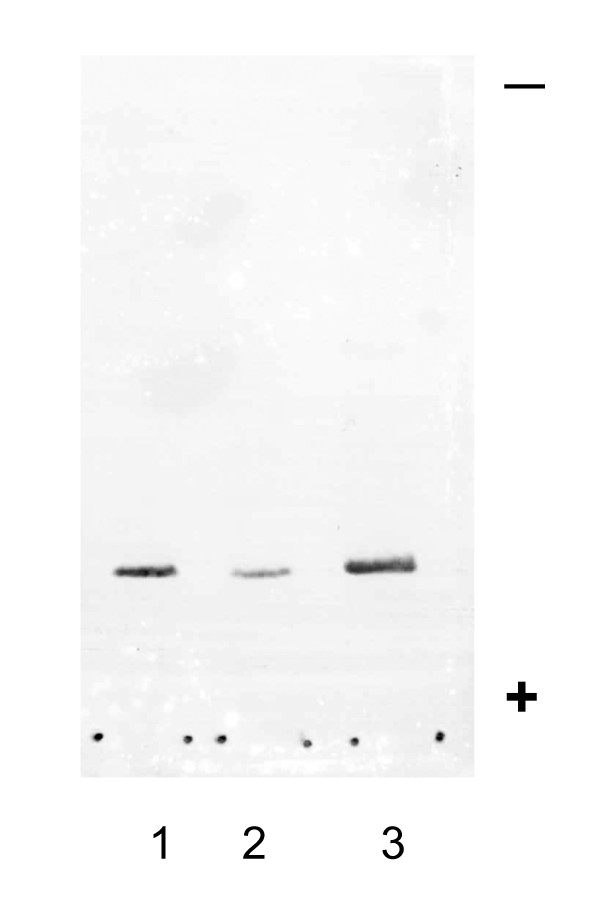
**Western blotting after SDS-PAGE**. A single band of CA-III from purified chicken CA-III (1), male WL-chicken hemolysate (2), and female WL-chicken hemolysate (3) is observed after staining by immunochemical method using anti-chicken CA-III serum. All bands had an approximate molecular weight of 28 kD. These data indicate that the antibody against chicken CA-III produced in the present study is specific to chicken CA-III.

Antibodies against chicken CA-III were produced in rabbits. The specificity of the antiserum was evaluated by double immunodiffusion. The antiserum to chicken CA-III formed a single precipitin line with crude chicken muscle extract and purified chicken CA-III, and the precipitin line fused completely (data not shown). Anti-chicken CA-III does not react with chicken CA-II [19] or canine CA-I, CA-II, or CA-III [9,20]. Figure 6 shows the results of western blotting for CA-III on purified chicken CA-III, male WL-chicken hemolysate, and female WL-chicken hemolysate. The molecular weight of these bands was approximately 28,000. Using the ELISA system developed in this study, 40 μg/mL of canine CA-III reacts only slightly with anti chicken CA-III, and the cross reactivity was 0.05%. By contrast, 40 μg/mL each of chicken CA-II, canine CA-I, and canine CA-II, did not react with anti-chicken CA-III. These data indicate that the antibody against chicken CA-III produced in the present study is specific to chicken CA-III.

### Measurement of CA-III

**Figure 7:**
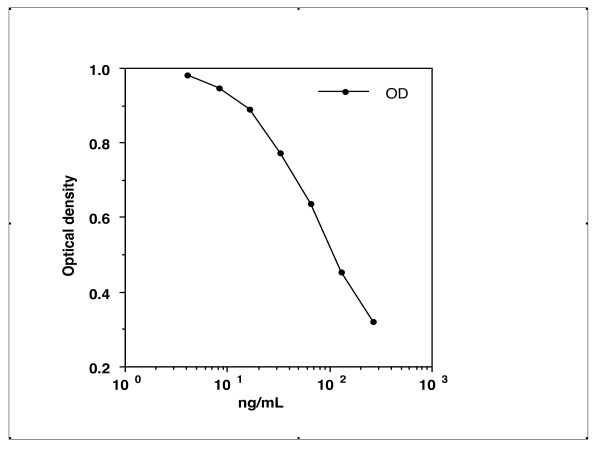
**Standard curves for chicken CA-III concentrations generated by use of the competive ELISA specific for chicken CA-III**. Typical standard curves were plotted for a range of CA-III solutions (2-400 ng/mL). The coefficients of variation (CV) for these solutions were as follows: 400 ng/mL, CV = 4.3%; 200 ng/mL, CV = 4.8%; 100 ng/mL, CV = 2.4%; 50 ng/mL, CV = 6.8%; 25 ng/mL, CV = 3.6%; 12 ng/mL, CV = 3.1%; 6 ng/mL, CV = 2.4%. Values were obtained by using a spectroscope at 405 nm.

Typical standard curves were plotted for a range of CA-III solutions (2-400 ng/mL) (Figure 7). The coefficients of variation (CV) for these solutions were as follows: 400 ng/mL, CV = 4.3%; 200 ng/mL, CV = 4.8%; 100 ng/mL, CV = 2.4%; 50 ng/mL, CV = 6.8%; 25 ng/mL, CV = 3.6%; 12 ng/mL, CV = 3.1%; 6 ng/mL, CV = 2.4%.

**Figure 8:**
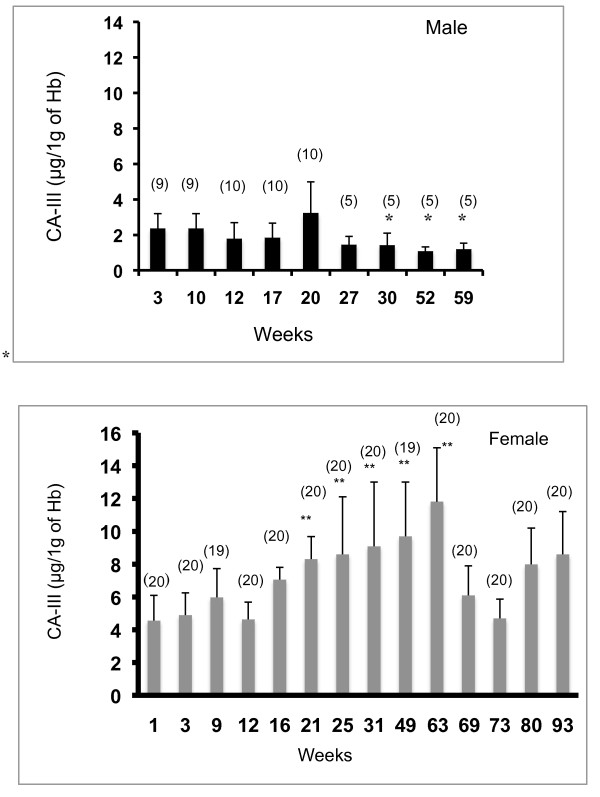
**Developmental changes in erythrocyte CA-III levels in male and female WL-chickens**. Means and standard deviations of erythrocyte CA-III concentrations (μg/g of Hb) in male (top) and female (bottom) WL-chickens. The number of samples is shown on the column. The CA-III level in erythrocytes of male WL-chickens did not change significantly from 3 to 27 weeks of age. The mean levels of CA-III in male WL-chicken erythrocytes at 30, 52, and 59 weeks of age were less than that at 20 weeks of age (p < 0.05). The levels of CA-III in female chicken erythrocytes (1 week old) did not change until 16 weeks of age. From 21 weeks of age, the CA-III levels in female WL-chickens increased significantly at 25, 31, 49, and 63 weeks of age. The level of CA-III in hemolysate from female WL-chickens peaked at 63 weeks of age. The CA-III then decreased gradually until 73 weeks of age and increased again until 93 weeks of age. : *: p < 0.05, **: p < 0.01.

The concentrations of CA-III in hemolysate samples from female WL-chickens (1-93 weeks old) and male WL-chickens (3-59 weeks old) were assayed using competitive ELISA, and the results (mean ± SD) for developmental changes are shown in Figure 8. The levels of CA-III in female chicken erythrocytes (1 week old) were 4.6 ± 1.5 μg/g of Hb, and this level did not change until 16 weeks of age. From 21 weeks of age, the CA-III levels in female WL-chickens increased significantly at 25, 31, 49, and 63 weeks of age (p < 0.01). The CA-III in female chicken erythrocytes approximately doubled between 1 and 21 weeks of age. The level of CA-III in hemolysate from female WL-chickens peaked at 63 weeks of age (11.8 ± 3.3 μg/g of Hb). The CA-III then decreased gradually until 73 weeks of age and increased again until 93 weeks of age (8.6 ± 2.6 μg/g of Hb). Simple linear regression analysis showed a significant association between the level of CA-III in erythrocytes and the egg-laying rate from 21 to 63 weeks of age in WL-chickens (p < 0.01).

The mean level of CA-III in erythrocytes from 3-week-old male WL-chickens was 2.4 ± 0.8 μg/g of Hb. This level was only half that measured in erythrocytes from female WL-chickens at the same age. The CA-III level in erythrocytes of male WL-chickens did not change significantly from 3 to 27 weeks of age. The mean levels of CA-III in male WL-chicken erythrocytes at 30, 52, and 59 weeks of age were less than that at 20 weeks of age (p < 0.05).

There was a statistically significant difference (p < 0.01) between CA-III levels in erythrocytes from female and male WL-chickens.

The plasma concentrations of CA-III in WL-chickens are shown in Table 1 (male) and Table 2 (female). The plasma concentration of CA-III in male WL-chickens did not change significantly between 3 and 60 weeks of age. The plasma concentration of CA-III in female WL-chickens was 60 ng/mL at 1 weeks of age and increased at 3 weeks of age but did not change significantly between 3 weeks and 80 weeks of age.

**Table 1:** The concentration of CA-III in the plasma from male WL-chicken from 3 weeks of age until 59 weeks olds.

Age (week)	n	CA-III (ng/mL)
		(mean ± SD)
3	8	58 ± 17
10	8	98 ± 22
12	7	107 ± 25
17	10	105 ± 37
20	10	88 ± 36
27	4	67 ± 34
30	5	70 ± 52
52	5	64 ± 9.0
59	4	88 ± 24

The plasma concentration of CA-III in male WL-chickens did not change significantly between 3 and 60 weeks of age.

**Table 2:** The concentration of CA-III in the plasma from female WL-chicken from 1 weeks of age until 93 weeks old.

Age (week)	n	CA-III (ng/mL)
		(mean ± SD)
1	16	60 ± 21*
3	15	141 ± 30
9	15	166 ± 30
12	15	174 ± 27
16	13	166 ± 40
21	10	141 ± 58
25	5	167 ± 53
31	5	159 ± 46
49	5	161 ± 39
63	10	161 ± 32
39	16	152 ± 43
73	13	146 ± 36
80	18	122 ± 30
93	7	105 ± 40*

The plasma concentration of CA-III in female WL-chickens was 60 ng/mL at 1 weeks of age and increased about twofold between 1 and 3 weeks of age. After the initial increase, the plasma CA-III level did not change significantly from 3 to 80 weeks of age.

*: p < 0.01.

Simple linear regression analysis did not show any significant association between the plasma level of CA-III and the egg-laying rate in female WL-chickens from 16 to 63 weeks of age (p > 0.5). There was a significant difference (p < 0.01) in plasma CA-III levels between female and male WL-chickens.

## Discussion

### Identification of chicken CA-III

In the present study, we developed a relatively simple procedure for purification of CA-III from chicken muscle. The purity of the CA-III was verified by SDS-PAGE and IEF-PAGE analyses. A clear partial amino acid sequence (NYPMA) from digested chicken CA-III was 100% identical to the carbonic anhydrase of cyanobacteria according to a FASTA search of the SWISS-PROT databank. The reported specific activities of equine and canine CA-III are 400 units/mg protein and 370 units/mg protein, respectively [5,9]. These specific activities are similar to the 410 units/mg protein measured for CA purified from chicken muscle in this study; taken together, these findings are consistent with the conclusion that the low-activity CA isozyme purified from chicken muscle in the present study is CA-III.

### CA-III levels in erythrocytes

This is the first description of CA-III levels in chicken erythrocytes. Chicken erythrocytes contain CA-II but not CA-I [21]. In the present study, it is apparent that chicken erythrocytes also contain CA-III. Erythrocytes from female chickens (i,e., 25 weeks old) contained about 1/22,000 as much CA-III as CA-II [19]. The level of CA-III in human erythrocytes was previously reported to be 147 ± 17 μg/g of Hb [22]. The level of CA-III in equine erythrocytes was previously reported to be 0.32 ± 0.11 μg/g of Hb [23], and the mean concentration of CA-III in canine erythrocytes was previously reported to be 4.9 μg/g of Hb [9]. The chicken erythrocyte CA-III level was therefore 29 times higher than the equine level and 2 times higher than the canine level but only 1/16 of the human level.

To confirm whether the level of CA-III in chicken erythrocytes changes during development, erythrocytes were analyzed for CA-III by ELISA. Both developmental changes and sex differences in CA-III levels in chicken erythrocytes were observed. In 21-63-week-old female chickens, erythrocyte CA-III levels were high and correlated with the high level of egg-production efficiency. Tanabe et al. [24] reported that positive correlations were observed between the egg production rate and luteinizing hormone (LH), progesterone, and testosterone concentrations in the chicken plasma.

Hoshino et al.[25] reported that corticosterone, thyroxine (T4), triiodthyronine (T3), and reverse triiodthyronine (rT3) concentrations in the chicken plasma increased during the molt. While, LH, estradiol, and progesterone concentrations declined during the molt, and these declines were coincident with the cessation of egg production. In the present study, the forced molt was induced at 64 weeks and stopped at 67 weeks. At the ages of 63, 69, 73, 80, and 93 weeks, the rates of egg laying were about 91, 2, 63, 91, and 82%, respectively.

The dog and mature rat exhibit sexual dimorphism in the expression of CA-III in the liver, but the horse, cow, and immature rat do not [17]. In the present study, chicken erythrocyte CA-III was sexually dimorphic. The erythrocyte CA-II level was previously reported to be much higher in female than in male chickens [19]. The erythrocyte CA-III levels in the female chickens decreased significantly from 69 to 73 weeks of age. The rates of egg laying were 2% at 69 weeks of age and 63% at 73 weeks of age, respectively. This suggests that the synthesis of CA-III in the erythrocytes at 69 and 73 weeks of age was affected by the forced molt. The synthesis of CA-III within the erythrocyte of laying chicken was probably control by these hormones. However, there was no evidence that these hormones stimulated the synthesis of the CA-III within the erythrocyte of laying chicken. Further investigations of physiological functions of CA-III in the erythrocytes of chicken are needed.

Everaert et al. [26] reported that injection of acetazolamide (ATZ), an inhibitor of CA, increased blood *P*co_2 _and decreased blood pH in both control and CO_2_-incubated chicken embryos. ATZ also increased blood bicarbonate concentrations in chicken embryos exposed to normal atmosphere and in day 12 embryos exposed to high CO_2_. They concluded that no single process (e.g., CO_2 _buffering by blood CAs) could explain the increase of both CO_2 _and HCO_3_^- ^in response to ATZ and hypothesized the existence of an isozyme other than CA-II in erythrocytes. Although CA-III shows low CO_2 _hydration activity, it has greater resistance to inhibition by ATZ [1]. In the present study, we were the first to find CA-III in chicken erythrocytes.

### CA-III levels in plasma

The human serum CA-III level was previously reported to be 99 ± 5 ng/mL [27]. The equine serum CA-III level was previously reported to be 18 ± 12 ng/mL [23], and the mean canine serum CA-III level was previously reported to be 17 ± 10 ng/mL (n = 116) [9]. In the present study, the plasma CA-III levels in 31-week-old female and 30-week-old male chickens were 159 ± 46 ng/mL and 70 ± 52 ng/mL, respectively.

Plasma was analyzed by ELISA to determine whether chicken plasma CA-III levels changed during development. Developmental changes and sex differences were observed in chicken plasma CA-III levels. The plasma CA-III level in female chickens increased about twofold between 1 and 3 weeks of age. After the initial increase, the plasma CA-III level did not change significantly from 3 to 80 weeks of age and did not correlate with the egg production efficiency. The plasma CA-III level was also not affected by the forced molt.

By contrast, the plasma CA-III level in 3-week-old male chickens was half that of females at the same age and remained stable throughout development.

## Conclusions

Developmental changes and sex differences were observed in CA-III concentrations in erythrocytes and plasma from WL-chickens. Simple linear regression analysis showed a significant association between the erythrocyte CA-III level and the egg laying rate in 16-63-week-old WL-chickens (p < 0.01).

## Competing interests

The authors declare that they have no competing interests.

## Authors' contributions

Sample collection design: TN and YT. Sample collection and processing: TN and DY. Survey design and implementation: TN, HO. Survey data entry: TN and KO. Analysis: TN, KO, and KA drafted the paper; the other authors helped to write the paper. All authors read and approved the final manuscript.
